# Performance optimization of LSCF/Gd:CeO_2_ composite cathodes via single-step inkjet printing infiltration

**DOI:** 10.1007/s10800-017-1066-1

**Published:** 2017-03-27

**Authors:** R. I. Tomov, Tom Mitchell-Williams, Chenlong Gao, R. V. Kumar, B. A. Glowacki

**Affiliations:** 1 Department of Materials Science and Metallurgy, University of Cambridge, 27 Charles Babbage Road, Cambridge, CB3 0FS UK; 2 Institute of Power Engineering, Warsaw, Poland

**Keywords:** Inkjet printing, Infiltration, Lanthanum strontium cobaltite ferrite, Doped ceria, Solid oxide fuel cells

## Abstract

**Abstract:**

The effect of solid oxide fuel cell cathode microstructure modification on its electrochemical activity is investigated. Inkjet printing infiltration was used to develop a nano-decoration pattern on the composite cathode scaffolds. Two types of composite La_0.6_Sr_0.4_Co_0.2_Fe_0.8_O_3−δ_:Ce_0.9_Gd_0.1_O_1.9_ cathodes with different volume ratios (60:40 and 40:60 vol%) were fabricated using inkjet printing of suspension inks. The electrodes were altered by single-step inkjet printing infiltration of ethanol-based Ce_0.9_Gd_0.1_O_1.9_ ink. After heat treatments in air at 550 °C the cathodes’ surfaces were shown to be nano-decorated with Ce_0.9_Gd_0.1_O_1.9_ particles (~20–120 nm in size) dispersed uniformly onto the electrode scaffold. The nano-engineered microstructure enhanced the active triple phase boundary of the electrode and promoted the surface exchange reaction of oxygen. Electrochemical impedance tests conducted on symmetrical cells showed a reduction in the polarization resistance of between 1.3 and 2.9 times. The effect was found to be more pronounced in the 60:40 vol% composite cathodes. Ageing of infiltrated electrodes up to 60 h in air revealed enhanced stability of gadolinium doped ceria nanoparticles decorated electrodes ascribed to the suppression of SrO surface segregation. This work demonstrated that single-step inkjet printing infiltration can produce reproducible performance enhancements and thus offers a cost-effective route for commercial solid oxide fuel cell infiltration processing.

**Graphical abstract:**

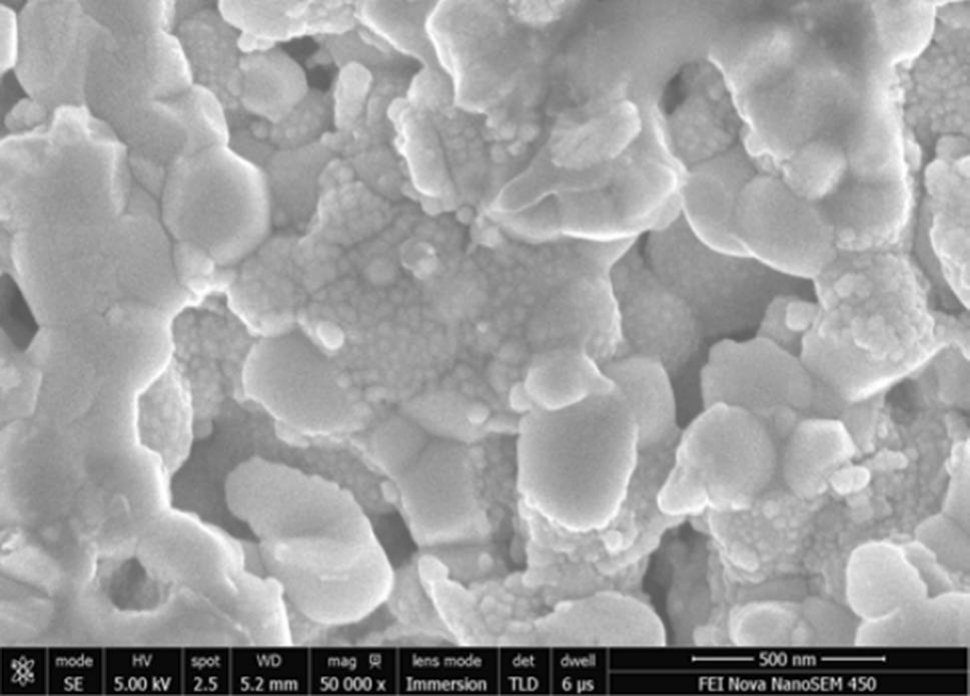

## Introduction

The drive towards commercialization of small scale (from sub-kW to several 10’s kW) Solid Oxide Fuel Cells (SOFCs) applications in the last decade has accelerated the research into intermediate-temperature SOFCs (IT-SOFCs). Lowering the operating temperature bellow 700 °C is envisaged to allow better long-term performance stability and wider choice of SOFC materials (e.g. cheaper metallic separators). It can allow faster start-up and ultimately reduce both the production and the operating costs of SOFCs [1, 2]. The high activation energy of the cathodic oxygen reduction reaction (ORR) at lower operating temperatures, which leads to a substantial decrease of the electrode reaction rates, is considered one of the main challenges for IT-SOFC applications. Lowering the operational temperature results in the reduction of the overall electrochemical performance due to increased Ohmic losses as well as electrode polarization losses. Hence, several strategies have been developed for successful implementation of IT-SOFC. One of them is based on lowering the Ohmic resistance by fabricating thinner electrolyte and/or substituting commonly used yttrium stabilized zirconia (8YSZ) with higher ionic conductivity material like gadolinium doped ceria (e.g. Ce_0.9_Gd_0.1_O_1.9_—CGO). The other is pursuing an increase of cathode and anode electrochemical activities through surface nano-engineering of the porous electrodes.

The performance of state-of-the-art commercial cathodes based on La_1−*x*_Sr_*x*_MnO_3−δ_ (LSM) is hindered by insufficient ORR activity and low oxygen ion conductivity at intermediate working temperatures. Thus La_1−*x*_Sr_*x*_Co_1−*y*_Fe_*y*_O_3−δ_ (LSCF) is preferred as a material of choice for working temperatures below 700 °C having high electronic and ionic conductivity as well as good catalytic activity for oxygen reduction reaction [3–7]. LSCF is a mixed ionic- electronic conductor (MIEC) belonging to the perovskite family, ABO_3_-type, in which both A and B sites can be partially or fully substituted. Partial substitution of La^3+^ for Sr^2+^ in LaCoO_3−δ_ was found to be accompanied by an increase in mean oxidation state of the cobalt ions and a significant increase in oxygen deficiency due to charge compensation [5, 6]. Substituting Fe on the B site gradually decreases the degree of ionic conductivity at lower temperatures [7] but also leads to a reduced thermal expansion coefficient (TEC) and hence better thermo-mechanical compatibility with CGO electrolytes [3, 6]. LSCF6428 (La_0.6_Sr_0.4_Co_0.2_Fe_0.8_O_3−δ_) is often chosen as a compromise offering high electronic conductivity (~340 S cm^−1^ at 550 °C [6]), high ionic conductivity (~1 × 10^−1^ S cm^−1^ at 800 °C in air [7]) and a lower TEC value (~15.3 × 10^−6^ K^−1^ at 373–873 K) [6]. Using isotopic exchange depth profiling, Esquirol et al. [8] reported that LSCF:CGO composite cathodes offer further improvement at temperatures below 650 °C. However, the structural stability of LSCF-based cathodes limits their widespread application. LSCF-based cathodes were found to suffer from substantial long-term degradation, typically at a rate of 0.05% per hour [9]. The strontium surface segregation and the coarsening of the cathode microstructure are often reported as possible degradation mechanisms for LSCF-based cathodes. As reported by Ding et al. [9] the combined effects of reduced surface stress and smaller surface charge results in SrO-terminated surfaces having lower energy than LaO-terminated surfaces thus promoting Sr segregation. The enrichment of Sr at the cathode surface leads to an increase in the cathode polarization losses through deactivation of ORR sites and decrease in surface activity [10]. Lowering the operational temperature is a straightforward way to alleviate such stability problem and to prolong the lifetime of the cell. Necessarily, the expected reduction of electro-catalytic activity needs to be counteracted by extending the TPB density and minimizing the polarization losses.

In recent years, significant progress has been made towards reducing the polarization losses and enhancing electrochemical activity and stability via infiltration of active precursors into the electrode scaffolds. Such infiltration procedures have been successfully implemented for both, anodes and cathodes, by various research groups [11–13]. Infiltration of fluorite-type materials such as doped ceria into LSCF was reported to increase TPB as well as promote surface exchange of oxygen [11]. Doped ceria is characterized by a high ionic conductivity, high surface exchange coefficient, high oxygen storage capability and good chemical compatibility with LSCF. The infiltration is usually a three-stage process involving the formation of mono-phase or composite scaffold in the first stage. The second stage is the infiltration of a catalytic or non-catalytic precursor. Inks are often tailored with suitable surfactants and complexing agents promoting the desired phase formation and morphology of the infiltrate product—continuous coating or nano-decoration with tailored particle size distribution. Often vacuum is implemented between multiple infiltration steps in order to increase the mass load of the infiltrate. An appropriate heat treatment is the required third stage which in some cases is performed after each loading step of the second stage. The infiltration procedures are predominantly performed on the lab scale by the use of microlitre syringes or pipettes. The process is cumbersome and slow, often resulting in a non-uniform ink distribution (both laterally and depth-wise), aggregation of nanoparticles in the case of high mass load infiltrations and waste of expensive precursors.

The infiltration of Gd_0.2_Ce_0.8_O_2_ into screen printed La_0.8_Sr_0.2_Co_0.5_Fe_0.5_O_3−δ_ was reported by Chen et al. [14] to significantly reduce the polarization resistance (*R*p) values as compared to the pure LSCF cathode from 0.22 to 0.06 Ω cm^2^ with 1.5 mg m^−2^ CGO loading at 750 °C. Nie et al. [15] performed infiltration of tape-casted LSCF6428 cathodes with aqueous nitrate solutions of Sm_0.2_Ce_0_.8O_1.95_ (SDC) precursors and glycine as a complexing agent. Impedance analysis of LSCF/SDC/8 mol% Y_2_O_3_−ZrO_2_/SDC/LSCF symmetric cells indicated dramatically reduced polarization losses, from a blank cathode polarization resistance value of 0.15 to 0.074 Ω cm^2^ for the infiltrated cathode at 750 °C. The infiltration of Ce_0.8_Gd_0.2_O_1.9_ sol ink into tape-casted LSCF6428 cathodes of symmetrical LSCF6428 /8YSZ/ LSCF6428 cells led to a reduction in cathode polarization resistance from 5.0 to 2.2 Ω cm^2^ at 700 °C as reported by Jeong Woo Yun et al. [16]. Enhanced performance and stability of LSCF cathodes when La_0.4875_Ca_0.0125_Ce_0.5_O_2−δ_ (LCC) was applied as a thin-film coating on the scaffold surface were reported by Liu et al. [17]. With 5 μL of 0.25 M LCC precursor was infiltrated into the LSCF cathode, the cathodic polarization resistance was reduced to 0.076 Ω cm^2^ from 0.130 Ω cm^2^ at 750 °C. Recently, Burye et al. [18] reported reduction of the cathode’s polarization resistance to 0.1 Ω cm^2^ at 540 °C by dual sequential infiltration of CGO and LSCF inks into a CGO porous scaffold. Similarly, the surface decoration of Co-free cathodes with SDC has been recently reported to enhance the oxygen reduction activity. Ding et al. [19] infiltrated SDC into LaNi_0.6_Fe_0.4_O_3−δ_ (LNF) cathode and observed a reduction of *R*p values of the infiltrated cathodes 5.1 times in comparison to the blank cathode. Multiple infiltrations of 3 M ink of SDC precursor into a double perovskite GdBaFeNiO_5+d_ (GBFN) electrode, as reported by Li et al. [20], formed a continuous three-dimensional decoration network producing 14 times reduction of the polarization resistance and showing lower activation energies compared to the blank cathode.

This study reports on the feasibility of inkjet printing technology for infiltration of porous SOFC electrodes. It explores optimization and stabilization of composite LSCF:CGO cathodes via single-step inkjet printing infiltration. Symmetrical cells based on CGO electrolyte and composite LSCF:CGO cathodes infiltrated with CGO inks were tested for enhanced performance and durability at temperatures between 550 and 650 °C. A single processing technique was chosen for both the fabrication of the electrodes and the infiltration itself—drop-on-demand (DoD) inkjet printing. The DoD inkjet printing is simple and cost-effective non-contact “wet” technique applicable on variety of surfaces including very thin fragile or non-even porous electrode supports. It can reproducibly dispense droplets in the range of *pL* to *nL* volumes at high rates (kHz). DoD inkjet printing allows excellent thickness and uniformity control and introduces the possibility of printing 2D and 3D patterns as well as delivering precursors into porous scaffolds with high accuracy. Infiltrated cathode nanostructures were created by consistently depositing nano-litre infiltrate droplets with micrometre spatial resolution onto the porous scaffold surface ensuring reproducibility between samples. Inkjet printing systems offer a wide scale of application: from experimental platforms working with customized inks, up to mass manufacturing systems that can print rapidly and competitively on industrial scale. The technology is cost-effective and environmentally friendly due to waste minimization of the expensive precursors, which is a critical issue for inks based on precious or rare earth metals. The production of anodes and electrolyte coatings with an in-house modified Domino print head was reported previously by Tomov et al. [21] and Wang et al. [22] using suspension inks. Wang et al. [23] deposited GDC electrolytes on NiO-8YSZ cermet anodes using sol–gel-based precursor solutions.

## Experimental

Cathode symmetrical cells were fabricated by inkjet printing of LSCF:CGO composite cathodes on each side of circular dense CGO pellets and infiltrated with Ce_0.9_Gd_0.1_(NO_3_)_3_ solution ink. A modified electromagnetic print head DOMINO with a 100 μm nozzle orifice was used for both procedures after jetting optimization for each of the inks.

### Ink preparation

#### Cathode composite inks

**Fig. 1:**
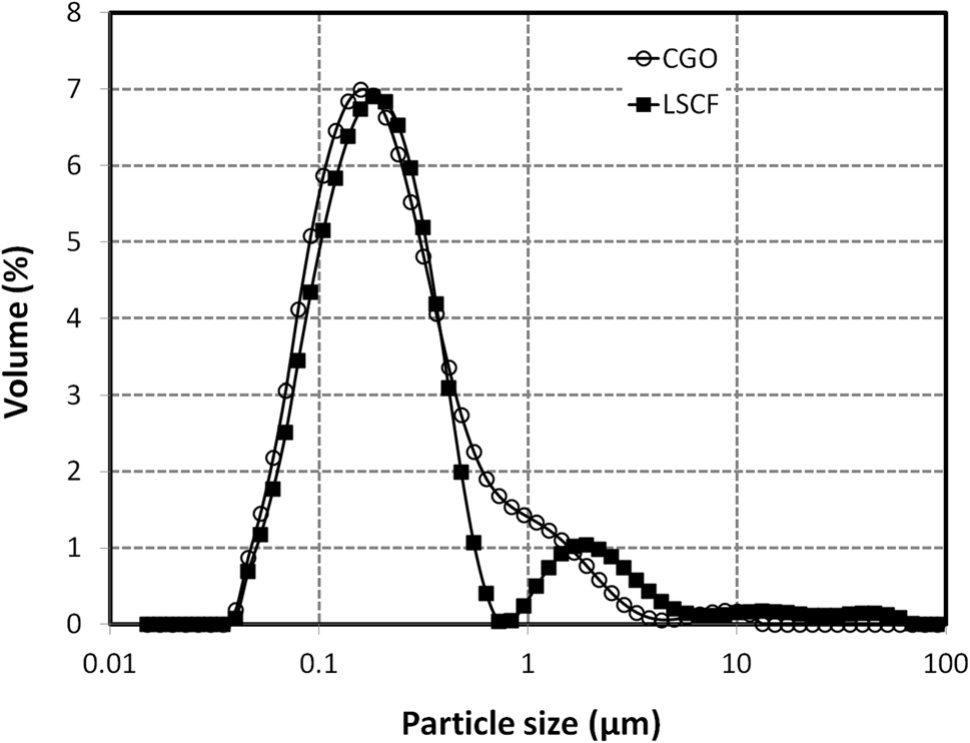
Particle size distribution of the primary CGO and LSCF inks diluted with methanol

Commercial La_0.6_Sr_0.4_Co_0.2_Fe_0.8_O_3_ (Fuel Cell Materials), denoted further as LSCF, Ce_0.1_Gd_0.9_O_2_ (Sigma-Aldrich), denoted further as CGO, and hydroxypropyl cellulose (Sigma-Aldrich) were used for the preparation of the cathode composite suspension inks. Two stable primary inks, containing LSCF and CGO, respectively, were made from commercial ceramic powders in a mixture with alpha-terpineol as a carrier and dispersants. The inks were produced by wet milling with 3YSZ milling balls in a planetary mill for various periods of time. Hydroxypropyl cellulose was used as a polymeric dispersant for ink stabilization. It not only provided steric stabilization of the suspension but also acted as a binder and a fugitive agent promoting the formation of a porous scaffold. The rheology of the inks was adjusted to the viscosity range required by the print head by varying the amounts of the diluting solvent. Methanol (reagent grade, Sigma-Aldrich) was chosen as a diluting solvent due to its high volatility allowing fast drying of the droplets. Terpineol (Sigma-Aldrich) was selected as a low vapour pressure carrier, inducing Marangoni flows counteracting the formation of coffee-ring stains. It also played the role of a natural dispersant of the oxide particles and had excellent miscibility with the dispersant and the diluting solvent. The particle sizes of the milled inks were analysed with MALVERN Mastersizer 2000 instrument (see Fig. 1). Bimodal distributions were observed for both inks with major peaks centred at ~0.15 μm particle diameter. The secondary peak was more pronounced for LSCF ink (at ~1.9 μm), while for the CGO ink the secondary peak had a maximum at ~1.0 μm. Composite suspension inks were produced by mixing and stirring both primary inks in the appropriate volume ratios and adjusting the viscosity levels with methanol. The inks were additionally filtered through 3 μm glass microfiber filters before being loaded into the nozzle compartments in order to separate the larger diameter “hard” agglomerates and the “tail” from the loaded part of the composite ink. The nozzle was observed to execute reproducible drop-on demand jetting without clogging the internal fluidic pathways of the assembly.

#### Infiltrate CGO ink

**Fig. 2:**
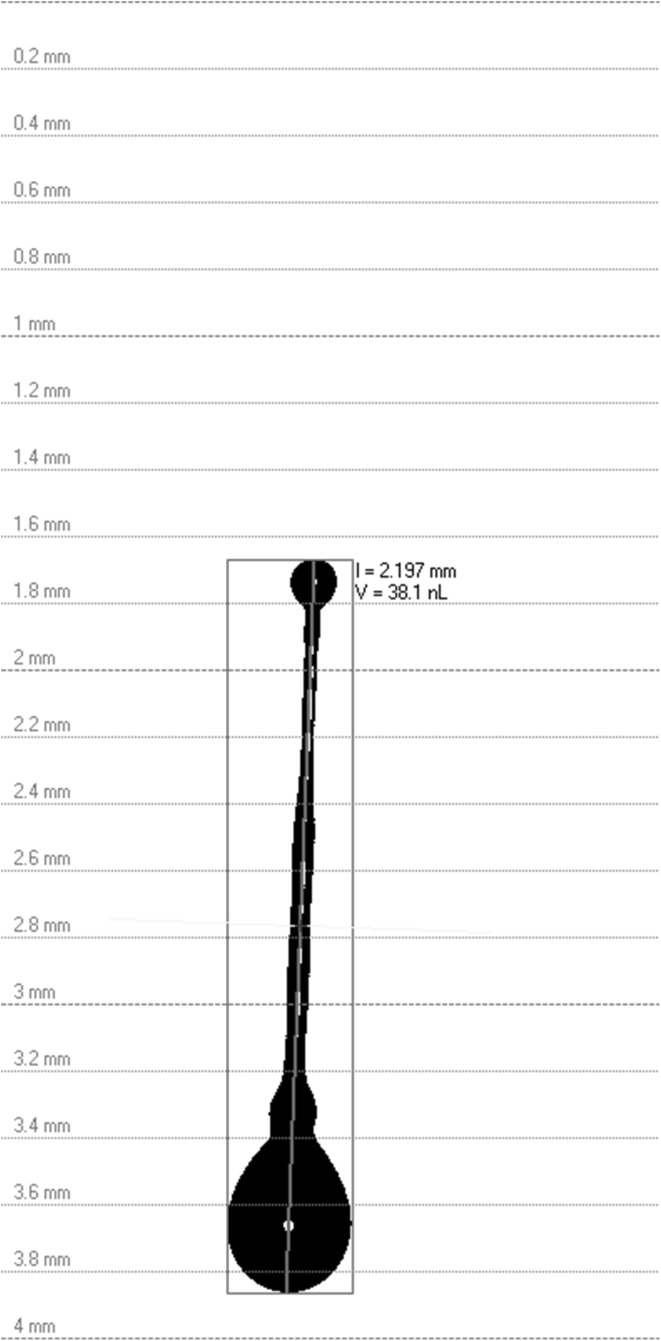
Optimized jetting behaviour of 0.5 M *EtOH*-based Ce_0.9_Gd_0.1_(NO_3_)_3_ ink under a pressure of 400 mbar and 220 μs nozzle opening time

Previous studies by Lou et al. [24] have shown that tuning the wetting properties of the infiltrate ink with organic solvents was an efficient way to improve the wettability on LSCF grains and enhance the uniformity of the nanoparticle distribution. Thus, Ce_0.9_Gd_0.1_(NO_3_)_3_ solution ink (1.0 M total metal concentration) was prepared by dissolving Ce(NO_3_)_3_·6H_2_O (99.5%, Sigma-Aldrich), Gd(NO_3_)_3_·6H_2_O (99%, Sigma-Aldrich) and citric acid—C_6_H_8_O_7_ [(99%, Sigma-Aldrich)] with metal ions:citric acid in a molar ratio of 1:1.5 in ethanol (*EtOH*). The precursor was further diluted in order to reduce the viscosity to suitable level as determined by the print head requirements (0.5 M). The uniform delivery of the ink by the moving print head requires precise knowledge of the drop velocity and the drop volume as well as avoidance of any delayed satellite drops formation. The use of a custom build integrated drop visualization system allowed us to optimize the jetting of the print head based on the variation of the nozzle opening time and operating pressure. Figure 2 illustrates optimized drop formation behaviour of the infiltrate CGO ink jetted under a pressure of 400 mbar and 220 μs opening time. The initial drop breaks into a main drop and one or more smaller drops after it is detached from the nozzle. For optimized printing parameters, the smaller drops soon merge with the main drop and form back into a single drop. Thus for suitably chosen jetting parameters no satellite drops will be deposited on the electrode. A detailed description of the drop jetting optimization as well as drop spreading optimization was reported previously [21–23, 25]. Further quantitative analysis also calculated the drop velocities and the drop volumes for the optimized jetting (drop volume *V* = ~38 nL, drop velocity = 3.7 m s^−1^).

### Symmetrical cell preparation

CGO powder, hydroxypropyl cellulose and *EtOH* were mixed and milled for 4 h. Following evaporation of the solvent in air at 120 °C, the powder was uniaxially pressed into pellets with 12.5 mm diameter under 195 kg cm^−2^ pressures. The pellets were fired using heating rate of 5 °C min^−1^ to 1400 °C and held for an hour which produced dense electrolyte substrates having diameter of ~11.6 mm and thickness of ~0.6 mm. Printing of the LSCF:CGO composite ink and CGO infiltrate ink was carried out using jetting parameters optimized in the drop visualization studies. X–Y movement speed of 40 mm s^−1^ was used for both the inks. A square array of droplets optimized for each ink drops spacing and overlapping was printed providing uniform coverage of the printed area. Detailed optimization studies on the printing patterns were published previously [21, 26]. The LSCF:CGO composite cathode coatings were printed onto both sides of the pellets in five sequential passes. Two different LSCF:CGO volume ratio cathodes—LSCF:CGO 40:60 vol% composite cathode (denoted further as 40:60) and LSCF:CGO 60:40 vol% composite cathode (denoted further as 60:40)—were used in order to evaluate the infiltration effect of Ce_0.9_Gd_0.1_(NO_3_)_3_ ink over different areas of LSCF grain surface available for decoration during the wetting of the LSCF:CGO scaffold. The cathodes were sintered in air at 1080 °C for 1.5 h at a ramping rate of 2 °C min^−1^. The final thickness of the LSCF:CGO cathodes was ~15 μm. Following an initial electrochemical impedance spectroscopy (EIS) testing, infiltrations of both sides of each cell were carried out with *EtOH*-based Ce_0.9_Gd_0.1_(NO_3_)_3_ ink. The ink was printed using an overlap of less than 10% between the droplets’ surface replicas in order to ensure uniform lateral distribution of the infiltrate ink. An intermediate heat treatment was applied after each deposition, increasing the temperature from ~20 to 120 °C. Such treatment led to the removal of the solvent within the scaffold thus aiding further infiltration steps. The samples were allowed to cool to room temperature before each deposition. Four infiltration steps were performed delivering 385 drops in a square pattern to each side of the symmetrical cells (~14 μL ink per side). We found this to be the loading limit achievable without any intermediate high-temperature sintering steps. The cells were then calcined at 700 °C in air for 1 h with 10 °C min^−1^ ramping and cooling rates. The loading of CGO nanoparticles was estimated by precision weight difference measurements between as-sintered and treated (infiltrated and calcined) samples. It was established that the as-described infiltration procedure produced loading levels of approximately 4 wt% of CGO nanoparticles with respect to the LSCF:CGO composite cathode scaffold. This was lower than the expected ~13 wt% which could be explained with the fact that some amounts of gelled ink residue were wiped out from the surface after the last infiltration step.

### Characterization

All symmetrical cells were characterized by EIS to determine their electrochemical performance. AC impedance measurements were performed with Solartron impedance analyser system (SI1260 + SI1287) using two electrode configuration. Silver mesh with an active area of ~0.78 cm^2^ and silver wires were used for contacting the electrodes. The EIS scans with AC amplitude of 10 mV were conducted at temperatures ranging from 500 to 650 °C at 50 °C intervals in ambient air. Impedance measurements were performed from 0.01 Hz to 100 kHz after 15 min of thermal equilibration at each temperature. The durability performance of the infiltrated cells was tested for periods between 20 and 60 h. The microstructure and the elemental composition of the cathodes were studied using high-resolution scanning electron microscopy FEGSEM (FEI Nova NanoSEM). A thin Pd coating was sputtered on the cross-section of the fractured samples in order to avoid charging effects during SEM analysis.

## Results and discussions

**Fig. 3:**
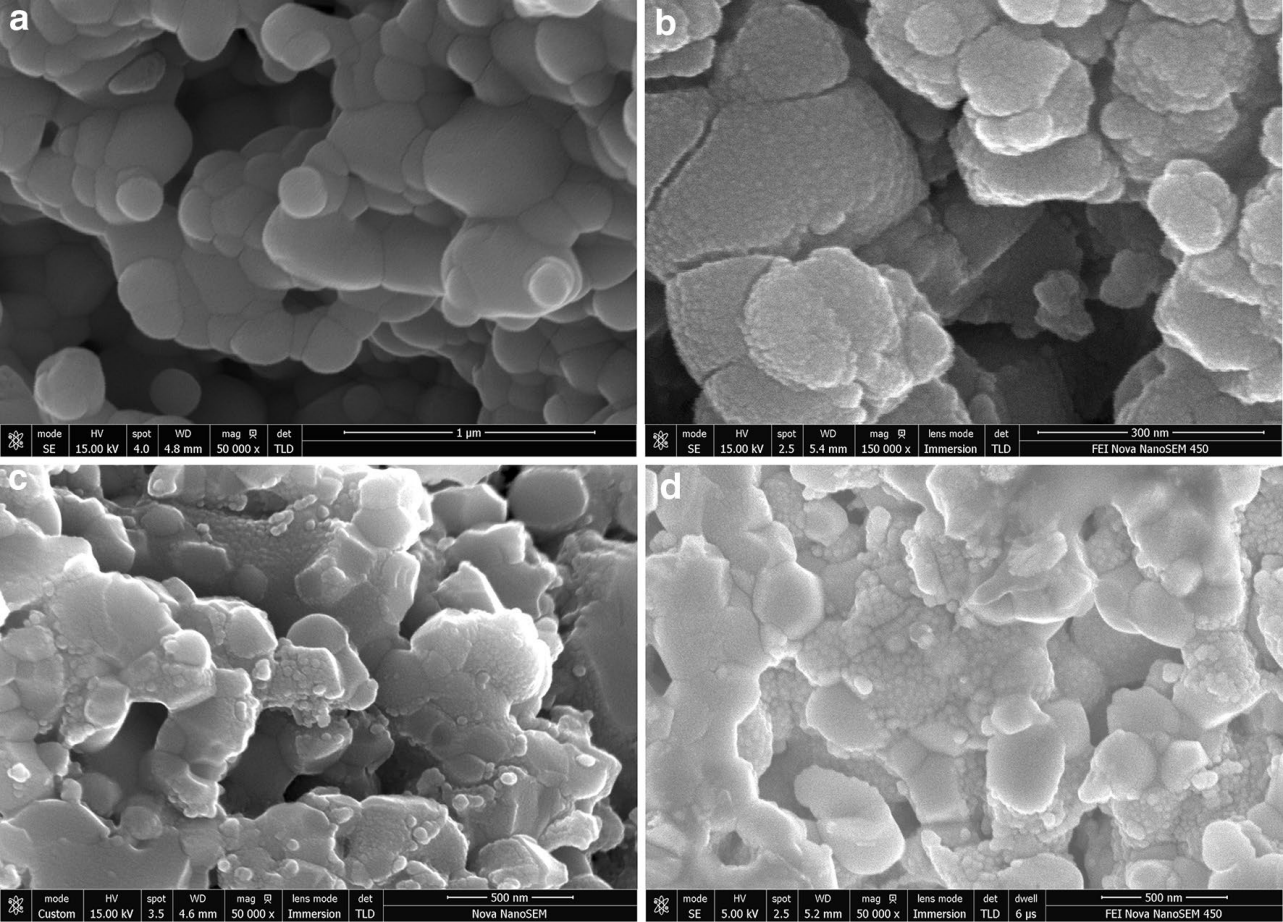
Scanning electron micrographs of fractured LSCF:CGO 40:60 vol% composite cathode **a** as-sintered at 1080 °C (*scale bar* 1 μm), **b** sintered at 1080 °C, infiltrated and calcined at 700 °C (*scale bar* 300 nm), c sintered at 1080 °C, infiltrated and calcined at 700 °C and aged for 20 h at 550 °C in air (*scale bar* 500 nm); **d** sintered at 1080 °C, infiltrated and calcined at 700 °C and aged for 60 h at 550 °C in air (*scale bar* 500 nm)

Figure 3a presents typical SEM cross-section image of 40:60 composite cathode scaffold surface as-sintered at 1080 °C. Porous backbone, uniform in terms of microstructure and grain size, with well interconnected LSCF and CGO grains (~500 nm in size) was observed. Figure 3b shows an identical LSCF:CGO composite cathode sintered at 1080 °C, infiltrated and calcined at 700 °C. It was found that after infiltration, the surface of the scaffold was uniformly covered with CGO nanoparticles. The size of the CGO nanoparticles was estimated to vary between 10 and 20 nm. Figure 3c, d illustrates the evolution of the LSCF:CGO scaffold microstructure after infiltration and ageing for different periods of time. The image in Fig. 3c was taken after ageing the infiltrated composite cathode at 550 °C in ambient air for 20 h. It revealed significantly reduced density of the CGO nano-decoration and an increase of CGO particle sizes varying between 20 and 60 nm. Testing and ageing for a longer period of 60 h were not found to alter substantially the size and the distribution of the CGO nanoparticles as shown in Fig. 3d. The images in Fig. 3a–d were collected from the fractured cross sections at locations near the electrode/electrolyte interface, confirming the good wettability of the scaffold with the *EtOH*-based Ce_0.9_Gd_0.1_(NO_3_)_3_ ink. Thus, the deposition of nano-scale ion-conducting phase was effectively extending the density of the active LSCF:CGO sites available for ORR which was expected to lead to superior electrochemical activity.

**Fig. 4:**
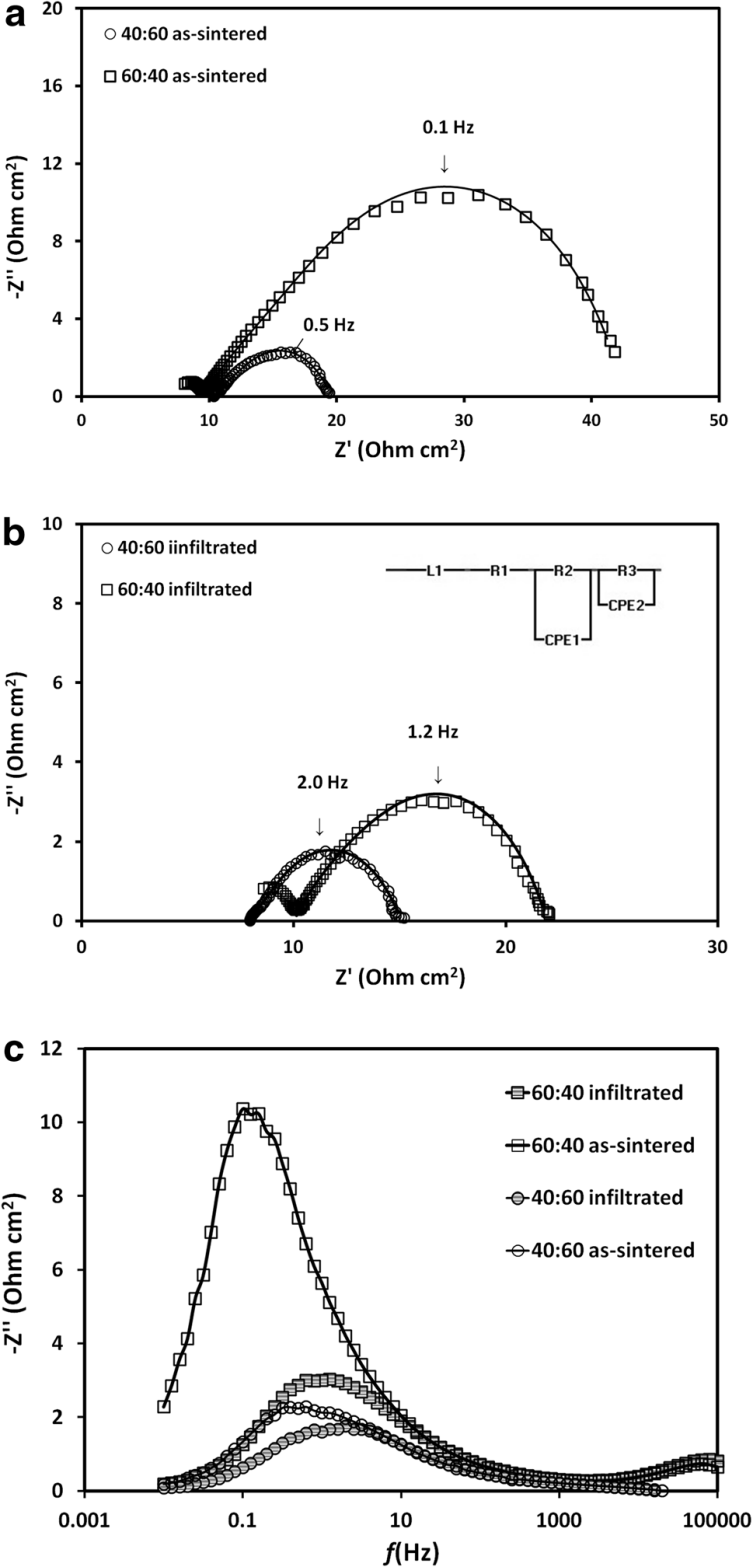
EIS data of CGO nano-decorated LSCF:GDC cathodes for both 40:60 and 60:40 LSC:CGO volume ratios measured at 550 °C in ambient air **a** Nyquist plot of as-sintered cathodes; **b** Nyquist plot of infiltrated cathodes and **c** Bode plot (imaginary impedance only) of as-sintered and infiltrated cathodes

In order to quantify the enhancement of the electrochemical activity of CGO-decorated cathodes towards ORR, two symmetrical cells with different vol% LSCF:CGO ratios were investigated by EIS. The EIS testing temperatures were varied between 500 and 650 °C. Figure 4a, b presents the EIS Nyquist plots of both composite cathodes measured at 550 °C in ambient air and showing results before and after CGO infiltration. The plots revealed depressed arcs similar to previously reported impedance behaviour of LSCF cathodes for ORR [14, 27] which was typically characterized by two overlapping arcs. Literature studies have assigned the high frequency EIS arc to the charge-transfer reaction at the LSCF:CGO interface and the low frequency EIS arc to oxygen surface exchange and/or dissociative oxygen absorption at the LSCF-air interface [28]. Although no clear separation between the low and high frequency arcs was observed in the temperature range between 500 and 650 °C, a fitting procedure with an equivalent circuit shown as inset in Fig. 4b suggested the existence of two arcs. In this circuit, the L1, R1, R2 (R3) and CPE 1(CPE 2) denote the inductance, ohmic resistance, electrode resistance and constant phase element, respectively. Fitted curves are presented in Fig. 4a, b.The activity of both types of electrodes was characterized by the interfacial polarization resistance *R*p, which was derived from sum of R2 and R3 (*R*p = R2 + R3), normalized by the electrode working area and divided by two. After the infiltration and formation of CGO nano-decoration, a significant reduction of *R*p values was registered for both type of LSCF:CGO cathodes.

The polarization resistances of the blank 40:60 cathode were measured as 0.94, 1.82, 4.67 and 15.07 Ω cm^2^ at 650, 600, 550 and 500 °C, respectively. The polarization resistances of the blank 60:40 cathode were 1.92, 6.05, 16.02 and 53.82 Ω cm^2^ at 650, 600, 550 and 500 °C, respectively. After the infiltration and formation of CGO nano-decoration, the polarization resistances for the 40:60 cathode were reduced to 0.48, 1.27, 3.55 and 8.03 Ω cm^2^ at 650, 600, 550 and 500 °C, respectively. The same infiltration and calcinations procedure led to a reduction of the polarization resistances for the 60:40 cathode to 0.74, 2.15, 5.74 and 18.61 Ω cm^2^ at 650, 600, 550 and 500 °C, respectively. The improvement in the polarization losses was substantially more pronounced for the 60:40 cathode. The 60:40 cathode provided larger LSCF grain surface available for nano-decoration with CGO nanoparticles compared to the 40:60 cathode. Hence, it had larger potential for a geometrical extension of the TPB and increase of the density of active sites for ORR. Figure 4c shows a frequency distribution of the imaginary part of the impedance. A shift of the position of the summit frequency (−Zʺ(*f*)_max_) from 0.1 Hz to 1.6 Hz was observed after the infiltration of the 60:40 cathode. For the 40:60 cathode, a similar shift from 0.5 to 2.0 Hz was registered. Both shifts were accompanied by lowering of the amplitude of −Zʺ(*f*)_max_. The relative increase of the summit frequency after infiltration with CGO was higher for the 60:40 cathode (16 times) compared to one for the 40:60 cathode (four times). This effect can be related to the larger number of CGO nanoparticles, created on the LSCF grain surfaces by the CGO infiltration, acting as promoters for the ORR reaction.

**Fig. 5:**
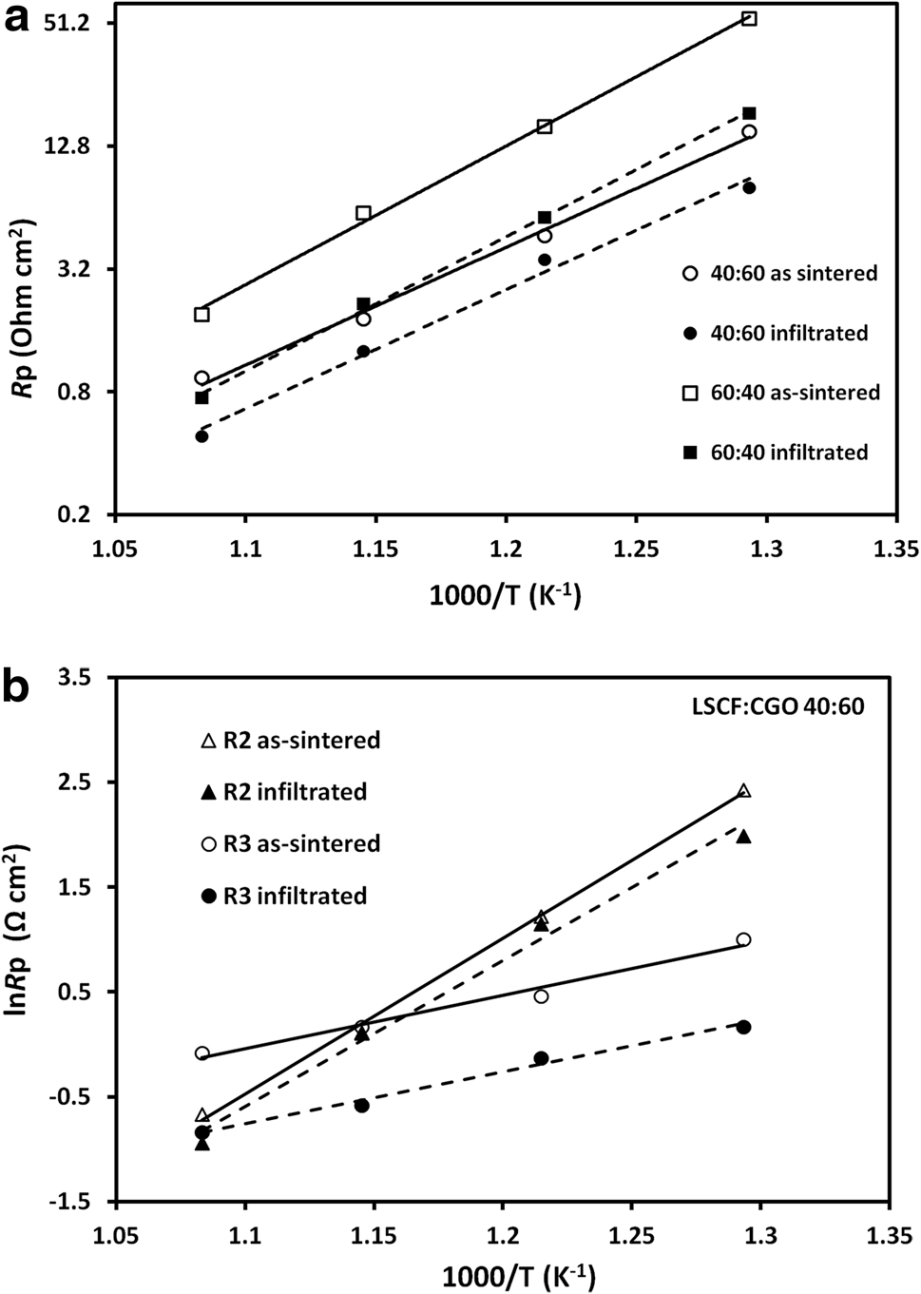
**a** Dependence of the polarization resistances vs testing temperature for as-sintered and CGO infiltrated LSCF:CGO composite cathodes. The inclined lines are a guide to the eye through the experimental data. **b** Arrhenius plots of the R2 and R3 components of the polarization resistance measured for LSCF:CGO 40:60 cathode—as-sintered and infiltrated. The *solid lines* represent as-sintered composite cathodes and *dashed lines* represent inkjet printing infiltrated cathodes

The variation of *R*p between 500 and 650 °C for both types of composite cathodes with and without infiltration is summarized in Fig. 5a. The decoration with CGO nanopartcles does not appear to change the activation energy of either of the composite cathodes significantly. Figure 5b compares the Arrhenius plots of the R2 and R3 parts of the polarization resistance measured for the 40:60 as-sintered and infiltrated cathodes. The results reveal that polarization resistance values for both electrochemical processes related to high- and low-frequency arcs were reduced to a different degree. For the infiltrated cathodes, the high frequency polarization resistance was found to dominate over the measured temperature span. The reduction of the overall polarization resistance appeared to be mainly due to a substantial promotion of low frequency-related processes with smaller effect on high frequency processes. Such tendency could be assigned to the relatively small loading of infiltrated CGO nanoparticles (~4 wt%) and their dispersed coverage leading to the lack of connectivity and percolation. Calculated activation energies for the as-sintered LSCF:CGO cathodes were 1.236 and 0.425 eV for R2 and R3, respectively. The infiltration treatment led to a small reduction of R 2 to 1.161 eV while the activation energy for R3 kept almost the same at 0.421 eV.

The ionic conductivity of CGO is higher than the ionic conductivity of LSCF at intermediate SOFC temperatures [14]. Thus forming a percolative CGO nanoparticle pattern on the surface of the LSCF grains can enhance TPB length creating additional oxygen ion-conducting paths and improving the electrochemical performance of the cathode. However, previous studies have shown that the addition of doped ceria can also accelerate the oxygen surface exchange rate [12, 29–32]. Changrong Xia at al. [30–32], using the electrical conductivity relaxation method, found that coating LSCF with Sm-doped ceria resulted in an increase by a factor of 10 in the surface exchange rate. The surface exchange coefficient of the SDC-coated LSCF was found to be dependent on the conductivity of doped ceria rather than the doping level of the SDC suggesting that doped ceria can provide additional free oxygen vacancies for the surface exchange reaction. LSCF was found to be much more active to oxygen exchange compared to that of doped ceria and the LSCF surface area exposed to the gas phase was in fact reduced by the infiltration as was the case in our experiment. Thus, it was assumed that the oxygen exchange process at LSCF/SDC/gas TPB sites was faster than that taking place on the pure LSCF surface. The effect was assigned to the additional free oxygen vacancies supplied by SDC. The effects of a single-step infiltration of small amount of Gd doped ceria observed in this work seemed to confirm the observations made by Changrong Xia at al [30–32]. Additional studies of *R*p of infiltrated and as-sintered composite cathodes as a function temperature and partial oxygen pressure are required in order to assess more precisely the limiting mechanisms in the high-frequency and low-frequency regions of the impedance spectra.

A direct comparison of the results reported by various groups on infiltration of SOFC electrodes appeared to be difficult due to the variation of reported figures of merit and the lack of convention on the measure of the infiltrate loading. There are many parameters having influence on the outcome of the infiltration procedure and they alter significantly between different studies—e.g. type of the scaffold (mono-phase or composite), thickness of the electrode, type of the ink (solvent and additives), number of the infiltration steps and the use of vacuum to assist the ink penetration between steps, calcinations temperatures (*T*_calc_) and durations, testing temperatures (*T*_EIS_). In order to evaluate the results of the present work, we have summarized the data reported by several groups on similar infiltrations of LSCF-based cathodes with doped ceria inks (see Table 1). The promotion factor as defined by Su et al. [33]—*ε* = *R*p_blank_/*R*p_inf_—was chosen as a figure of merit where *R*p_blank_ were the polarization resistance values of the blank cathodes and *R*p_inf_ were the polarization resistance values of the infiltrated cathodes. As shown in Table 1, the value of ε varies with the temperature and the type of infiltration procedure between 1.30 and 3.66. An exception of this trend is the result reported by Zhao et al. [34] on the infiltration of one-dimensional La_0.8_Sr_0.2_Co_0.2_Fe_0.8_O_3−d_ nanorod-based cathode infiltrated with Ce_0.8_Gd_0.2_O_1.9_ precursor. A significantly greater reduction in the polarization resistance (*ε* = 141.2) was reported illustrating the importance of the cathode microstructure. The nanorod-based scaffold in this case provided an optimized “one-dimensional” porosity allowing easier ink penetration and hence higher loading limit for the CGO precursor. The values of ε measured in this work varied between 1.31 and 2.90 with the lowest *R*p_inf_ = 0.48Ω cm^2^ measured for the 40:60 cathode at 650 °C. These values were comparable to the results reported by other research groups [14–16] and were achieved with almost three orders of magnitude lower ink expenditure (μl vs. ml) in a single-step infiltration procedure without intermediate high temperature calcinations.

**Table 1:** Improvement factor (ε) comparisons of doped ceria infiltrated LSCF:CGO cathodes at varying temperatures

Source	Infiltrated ink	Scaffold	Solvent and additives	T_calc_, °C	N_inf_^$^	T_EIS_, °C	*R*_p−blank_, Ω cm^2^	*R*_p−inf_, Ω cm^2^	ε	Loading level (method of loading)
Chen et al. [14]	Gd_0.2_Ce_0.8_O_2−x_	La_0.8_Sr_0.2_Co_0.5_Fe_0.5_O_3−δ_	Not specified	800	4	600	5.40	1.6	3.37	1.5 mg cm^2^(not specified)
						750	0.22	0.06	3.66	
Nie et al. [15]	Sm_0.2_Ce_0.8_O_1.95_ 0.25 M mol L^− 1^	La_0.6_Sr_0.4_Co_0.2_Fe_0.8_O_3 −δ_	Water propanol glycine	900	1	650	1.09	0.44	2.47	10 ml ink per electrode – (microliter syringe)
						700	0.40	0.17	2.35	
						750	0.150	0.074	2.02	
						800	0.064	0.041	1.05	
Yun et al. [16]	Gd_0.2_Ce_0.8_O_2−x_*	La_0.6_Sr_0.4_Co_0.2_Fe_0.8_O_3−δ_	Distilled water	700	1	600	53.8	29.50	1.80	(Not specified)
						700	5.00	2.20	2.27	
						800	0.45	0.30	1.50	
Zhao et al. [34]	Gd_0.2_Ce_0.8_O_2−x_ 0.25 M mol L^− 1^	La_0.8_Sr_0.2_Co_0.2_Fe_0.8_O_3−δ_ nanorods	Ethanol de-ionized water	800	4	650	14.12	0.10	141.2	160 μl per electrode 40 μL per infiltration step (microliter syringe)
This work	Gd_0.1_Ce_0.9_O_2−x_ 0.5 M mol L^−1^	La_0.6_Sr_0.4_Co_0.2_Fe_0.8_O_3−δ_/Gd_0.1_Ce_0.9_O_2−x_ (40:60)	Ethanol citric acid	700	1	500	15.07	8.03	1.87	14 μl ink per electrode 4 wt% 0.64 mg cm^−2^ (inkjet printing)
						550	4.67	3.54	1.31	
						600	1.82	1.27	1.43	
						650	0.93	0.48	1.93	
		La_0.6_Sr_0.4_Co_0.2_Fe_0.8_O_3−δ_/Gd_0.1_Ce_0.9_O_2−x_ (60:40)								
						500	53.81	18.61	2.89	
						550	16.01	5.74	2.78	
						600	6.04	2.15	2.80	
						650	1.92	0.74	2.56	

*CeO_2_ colloidal dispersion mixed with (Gd(NO_3_)_3_)·6H_2_O ink

^$^N_inf_ - number of infiltrations

**Fig. 6:**
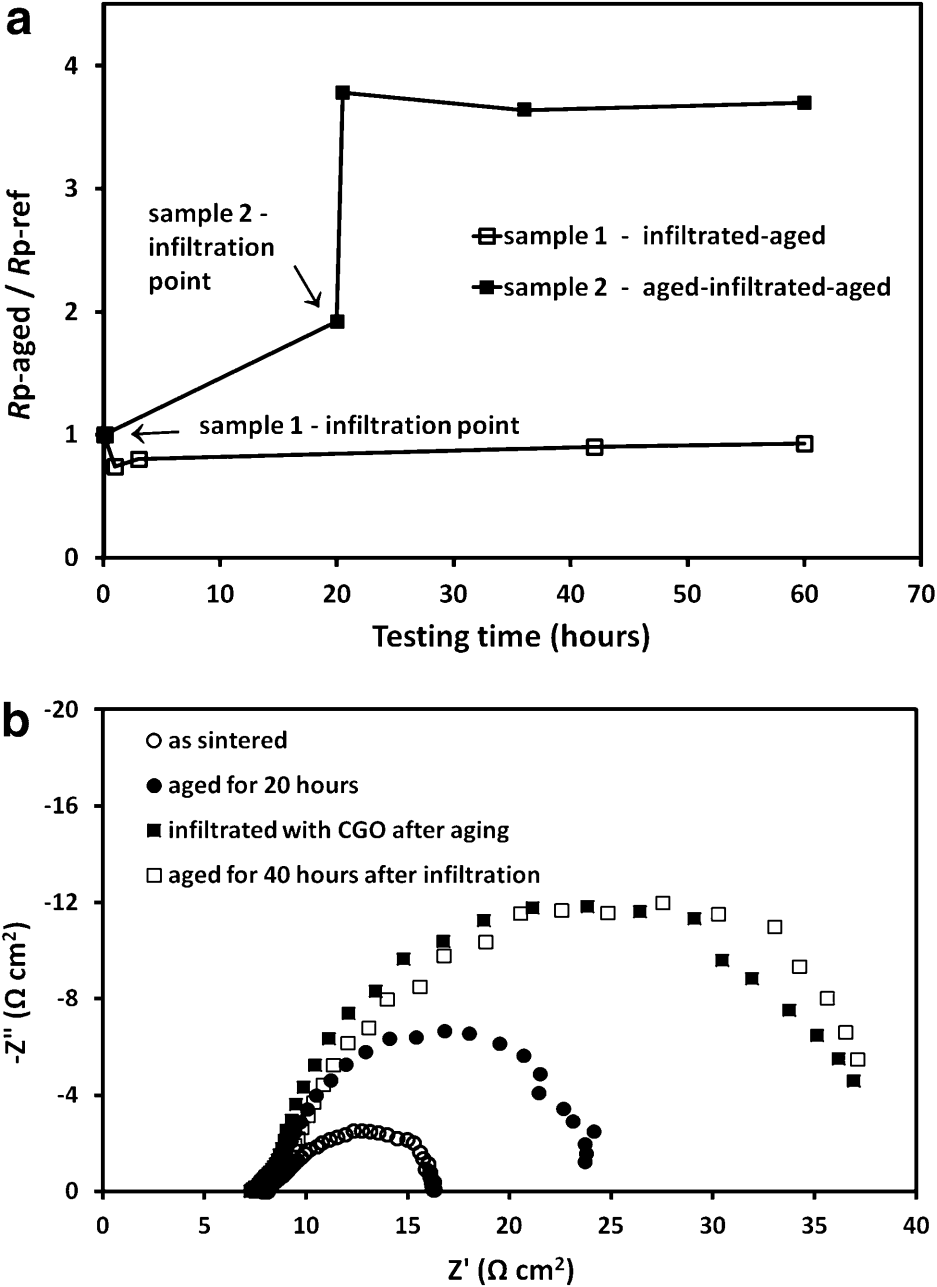
(**a**) *R*p_-aged_/*R*p_-ref_ ratio for 40:60 LSCF:CGO cathodes with different infiltration sequence during prolonged testing in ambient air at 550 °C (timing of the infiltration treatments is indicated by *arrows*); (**b**) EIS spectra of the cathode taken at four sequential processing stages—(i) as-sintered; (ii) after the first 20 h of ageing, (iii) after the infiltration and firing at 700 °C, (iv) after an additional 40 h of ageing

Prolonged testing in ambient air at 550 °C of as-sintered and infiltrated samples confirmed that the CGO infiltration not only reduced the polarization resistances but also enhanced the performance durability. A summary of the results is shown in Fig. 6a illustrating the time variation of the ratio between the *R*p values of 40:60 cathodes aged for different periods (*R*p_-aged_) with respect to the initial reference values of the blank cathodes before infiltration (*R*p_-ref_). The blank as-sintered cathodes experienced accelerated degradation rates during the first 20 h of testing (sample 2 in Fig. 6a). In contrast, the cathode infiltrated shortly after the initial testing (sample 1) showed negligible deterioration during 60 h of testing. In order to explore the role of the Ce_0.9_Gd_0.1_(NO_3_)_3_ solution ink infiltration on a pre-aged (partially degraded) cathode, sample 2 was infiltrated and calcined at 700 °C after the initial 20 h of testing. Contrary to the observed behaviour in sample 1, the *R*p values rose sharply after the infiltration; however, any further degradation was suppressed. The Nyquist spectra at four of the testing stages for sample 2 are presented in Fig. 6b.

Although the exact nature of LSCF cathodes degradation is not yet fully established, strontium surface segregation and the coarsening of the cathode microstructure are often reported as possible degradation mechanisms [9, 10, 35, 36]. The study by Zhang [36] concluded that the inter-diffusion of elements occurring during cathode high-temperature firing can lead to a destabilization of the compound. Long-term ageing conditions and segregation of SrO onto the LSCF surface were also correlated with the existence of surface contaminations and defects. Ding et al. [9] reported that reduced surface stress and smaller surface charge resulted in SrO-terminated LSCF surfaces and suggested introducing compressive strain as a way to suppress strontium oxide segregation. In this study, the SEM micrographs taken at different stages of prolonged testing (see Fig. 3b–d) showed no significant scaffold grain growth or CGO particles agglomeration at 550 °C. Thus, the performance stabilization of the composite LSCF:CGO cathodes after infiltration was assigned to the suppression of strontium surface segregation. Firstly, CGO has lower thermal expansion coefficient than LSCF. Hence, the CGO nano-decoration is expected to exert locally compressive strain inhibiting SrO segregation at the intermediate operating temperatures. Secondly, we assume that the infiltrate ink can effectively remove already segregated SrO and other contaminants from the aged LSCF surface due to the ink acidity. The detrimental effect of the infiltration on the performance of the pre-aged sample (sample 2) can be related to the amount of SrO segregated on the scaffold surface and effectively dissolved by the ink during the infiltration. One should also consider the possibility of doping the CGO nano-decoration with the dissolved by the ink SrO. According to Jaiswal et al. [37] doping ceria with Sr at levels higher than *x* = 0.05 (Ce_1−*x*_Sr_*x*_O_2−*x*_) leads to a formation of SrCeO_3_ besides the major fluorite phase. Consequently, the ionic conductivity decreases gradually with increasing x value due to the increased probability of formation of neutral associated defect pairs. Hence, we speculate that in this case, the dissolved Sr dopes the nano-CGO particles and reduces their ionic conductivity thus contributing to the observed cathode degradation after the infiltration of sample 2.

## Conclusions

Composite LSCF/CGO cathodes were fabricated and infiltrated with CGO via DoD inkjet printing. The infiltration was done in a single-step infiltration procedure without vacuum treatment or intermediate high temperature calcinations. The relatively low infiltration loading (~4 wt%) and the formation of highly ion-conducting CGO nano-decoration led to substantially enhanced cathode electrochemical performance. The infiltrated cathodes achieved promotion factors comparable to previously published data at significantly reduced expenditure of ink. Composite cathodes decorated with CGO nanoparticles showed stable operation at 550 °C for 60 h assigned to the suppression of SrO segregation by the CGO nano-decoration. Evidently, 60 h of durability testing is insufficient compared to the expected lifetime of a commercial SOFC. Nevertheless, the reported results are encouraging and prompting further tests on larger commercial cells for reasonably longer ageing periods. DoD inkjet printing infiltration was demonstrated to be feasible and cost-effective technology for nano-engineering of IT-SOFC electrodes. The combination of high speed of printing, accuracy of drops delivery, no wastage of ink and availability of industrial multi-nozzle systems presents an opportunity for scaling up the inkjet printing infiltration to a commercial level SOFC technology.
